# Biomechanics, muscle modeling, and the elevated bite force and tooth stress of piranhas

**DOI:** 10.1007/s00114-026-02071-w

**Published:** 2026-02-09

**Authors:** Steve Huskey, Keegan Fletcher, Matthew Kolmann, Serena Seiler, Gabrielle Kitchen, Devya Hemraj-Naraine, Ben Dinan, Mark W. Westneat

**Affiliations:** 1 Department of Biological Sciences, Western Kentucky University, Bowling Green, KY 42101 USA; 2 Department of Biology, University of Louisville, Louisville, KY 40292 USA; 3 College of Veterinary Medicine, Auburn University, Auburn, AL 36832 USA; 4 Center for the Study of Biological Diversity, University of Guyana, Georgetown, Guyana; 5 School of Engineering and Applied Sciences, Western Kentucky University, Bowling Green, KY 42101 USA; 6 Department of Organismal Biology and Anatomy, University of Chicago, Chicago, IL 60637 USA

**Keywords:** Characiformes, Feeding, Jaws, Neotropics, Piranhas, Serrasalmidae

## Abstract

The bite forces of vertebrate jaws and the application of those forces on a pointed or bladed tooth edge are crucial biomechanical parameters for any feeding event. Piranhas (fish family Serrasalmidae) exhibit powerful bites facilitated by large, multipennate, jaw muscles and high-efficiency lever mechanics, often accompanied by razor sharp teeth. Small animals like piranhas are often capable of impressive performance when viewed in a size-specific framework, yet the bite force capacity and tooth stress properties of piranhas have not often been measured or modeled. Here, we used morphometrics, computational biomechanical modeling, LiDAR teeth scanning technology, and comparative methods to explore the morphological underpinnings of the feeding mechanics in 11 species of piranhas representing disparate feeding guilds. Results show that piranhas produce highly variable tooth-specific bite forces (from < 1 N to almost 140 N) that yield elevated tooth tip stresses (from ~ 10 to > 440 MPa) for rapid removal of tissue from their prey. Our central conclusions are that some piranhas have mass-specific bite forces that exceed 1200 N/kg which greatly surpass large crocodylian, dinosaurian, and mammalian bite capacities, and that the greatest size-standardized bite forces and tooth stresses are found in an ectoparasitic species and omnivorous piranhas. Given the early-branching phylogenetic position of some of these omnivorous species, we conclude that diversification of the piranha feeding mechanism arose early in the evolutionary history of the clade.

**Supplementary Information:**

The online version contains supplementary material available at 10.1007/s00114-026-02071-w.

## Introduction

The evolution of the gnathostome jaw is a key innovation in the diversification and continuing success of vertebrates over the last 425 million years (Romer 1967; Brazeau and Friedman 2015; Johanson et al. 2019). Jaws and associated dentition provide the necessary hardware to be an effective predator, that is, to subdue, kill, and process prey (Wainwright 1988; Meers 2002). Likewise, a more forceful bite permits predators to tackle larger and more robust prey (Wainwright 1988; Clifton and Motta 1998). For these reasons, measuring feeding performance, frequently quantified as bite force, has a direct link to our understanding of an individual’s fitness and role in its ecological community (Arnold 1983; Grubich et al. 2012; Gignac and Erickson 2014).

Bite force has been quantified by numerous studies on both extinct and extant vertebrates (Erickson et al. 2003; Huber et al. 2006; Herrel et al. 2014; Huby et al. 2019). For example, a study conducted on *T. rex* bite indentations of fossilized skeletal remains revealed an estimated bite force ranging from 6,410 to 13,400 N (Erickson et al. 1996). One of the earliest (and now extinct) jawed vertebrates, *Dunkleosteus terrelli*, may have had one of the most forceful bites with bite forces measuring upwards of 4,400 N (Anderson and Westneat 2007). This bite is greater than many extant mammalian predators like the bone-crushing spotted hyena, *Crocuta crocuta* (3,500 N; Binder and Van Valkenburg 2000; Tanner et al. 2008), demonstrating that since the earliest advent of jaws, vertebrates have been pushing the envelope of feeding performance. In extant fishes, bite force has been examined in sharks and rays (Huber 2004; Huber et al. 2005, 2006; Mara et al. 2010; Kolmann et al. 2023), as well as in several teleosts (for examples, see Herrel 2002; Maie et al. 2009; Copus and Gibb 2013; Habegger 2017; De Meyer et al. 2018; Kaczmarek and Gidmark 2020). These high bite forces are impressive raw force values and yield high tooth stresses when applied to a prey item by the tips of the teeth, yet these animals are large and so the mass-specific bite force, in N of bite force per kg of body mass, is not among the highest values in animals. These large organisms fall short of the capabilities of the black piranha, *Serrasalmus rhombeus*, where a 1.1 kg specimen generated a bite force of 320 N (Grubich et al. 2012), which was the most forceful bite, relative to body mass, recorded for vertebrates. While *S. rhombeus* has the greatest in vivo bite force of the piranhas measured to date, many other species in the family Serrasalmidae also exhibit notable bite performance (Huby et al. 2019).

Piranhas and pacus (Family Serrasalmidae) are a monophyletic family of South American characiform fishes divided into three clades: Colossomatinae, Myleinae, and Serrasalminae, according to morphological and molecular data (Cione et al. 2009; Thompson et al. 2014; Kolmann et al. 2021). Whereas herbivorous and omnivorous pacus constitute the entirety of the colossomatine and myleine clades, piranhas, and their sister lineage, *Metynnis* (silver dollars or pacucitos) comprise the clade Serrasalminae (Thompson et al. 2017; Mateussi et al. 2020; Kolmann et al. 2021). Serrasalmids consume an impressive array of foods: from fruits, seeds, and insects in pacus, to scales in *Catoprion mento*, and mixed diets of plant matter, fins, fishes, and invertebrates in piranhas (Nico and Taphorn 1988; Ferreira et al. 2014; Kolmann et al. 2021). Rather than employ cranial kinesis or suction feeding like many other fishes, serrasalmids use biting as their primary mode of prey capture (Huby et al. 2019), which nonetheless allows them to exploit an exceptional variety of foods.

**Fig. 1:**
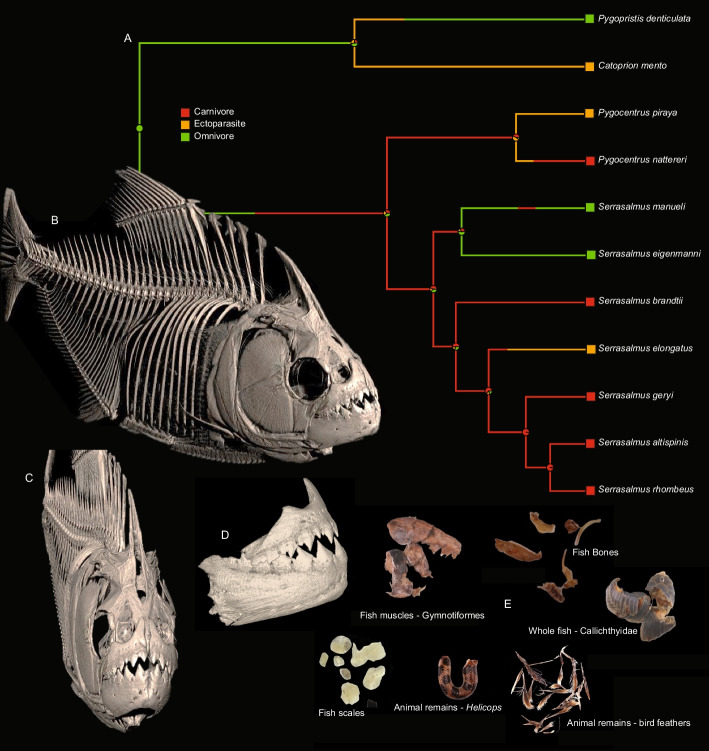
Skeletal and dental anatomy of a black piranha, *Serrasalmus rhombeus*, (ANSP 195066) obtained through micro-computed tomography data. **A** Ancestral state estimation of diet evolution in piranhas using stochastic character mapping. Based on diet information and phylogeny from Kolmann et al. 2021. **B** Lateral view of black piranha whole body. **C** Anterior view of black piranha. **D** Lateral view of the jaws. **E** Gut contents showing dismembered food remains (photos: D. Hemraj-Naraine). Osteological images rendered with CTVox (Bruker Softwares) and 3DSlicer (Kikinis et al. 2014). Scan parameters: Bruker SkyScan 1173, 35um, 70kV 114uA, 1 mm Al filter

Piranhas can achieve powerful bite forces by having relatively massive adductor mandibulae muscles (Alexander 1964; Datovo and Castro 2012), constituting up to 2% of their total body mass (Grubich et al. 2012), and jaw lever mechanics that significantly amplify force (Grubich et al. 2012; Huby et al. 2019). The adductor mandibulae complex is responsible for the force generated during jaw closing; therefore, powerful adductor mandibulae muscles and robust jaw bones are required for any fish that relies on biting for prey capture and processing. Fish jaw function is often modelled using lever and linkage mechanics (Westneat 2003; Huber and Motta 2004; Habegger et al. 2006), with jaw bones transferring muscle forces to the row of mandibular teeth used by predators to engage directly with their prey (Fig. 1).

Tooth shape is also variable among serrasalmids; most piranhas have seven tricuspid teeth on the lower and upper jaw (dentaries and premaxillae, respectively) with incredibly sharp edges and fine serrations (Huskey 2017; Huskey et al. 2020, Supplemental Materials, Figure S1:A) that allow them to excise chunks of fins and/or flesh from larger prey (Goulding 1980; Machado-Allison and Garcia 1986). These teeth interlock in a peg-and-socket fashion, with the cusps of the teeth providing insertion points for the neighboring tooth (Shellis and Berkovitz 1976; Kolmann et al. 2019).

Previous studies have revealed the magnitude of the bite forces produced by piranha jaws (Grubich et al. 2012; Huby et al. 2019; Lowe et al. 2023), as well as the functionality of their dentition (Kolmann et al. 2019), but an important next step is to reconcile bite force over the tooth surface area to demonstrate the maximum bite stress produced when the jaw’s forces and teeth meet a prey item. Force is quantified as mass multiplied by acceleration. Given this, imagine a 25 kg bag of grain resting atop your foot, accelerated by gravity to produce 245 Newtons. You would not feel discomfort because the bag conforms to the shape of your foot and the force is distributed over a large surface area. But stand a steak knife atop your foot with the bag of grain on top of the knife and the force per unit area, the *stress,* has increased by orders of magnitude (leading you to quickly adjust the situation). This analogy highlights the importance of combining bite force with a sharp cutting tooth edge in piranhas for removing pieces of their prey.

Bite forces interact with the dentition to achieve the stresses that damage and dismantle prey (Erickson et al. 2012, 2014; Gignac and Erickson 2015). Delivering high bite stress is how animal teeth create damage when biting their prey, and studies modeling relative tooth stress have shown its importance in the degree to which teeth are functionally homodont or heterodont (Cohen et al. 2023). For species with vast numbers of teeth (e.g., sharks with typically 50–300 functional teeth) or with blunt teeth (e.g., crocodylians with 30–55 peg-shaped teeth on the lower jaw and tyrannosaurs with 25–30 banana-shaped teeth on the lower jaw), their powerful bite forces are diluted by the cumulative surface area of their combined dentition, resulting in high bite forces but significantly reduced tooth stresses. Once these forces are rectified over the substantial surface area of their teeth, and scaled to the body mass of the predator, mega-predators like tyrannosaurs are likely quite a bit lower than piranhas when considering bite stress. For example, the black piranha is known to generate 35-times its own body mass in bite force (Grubich et al. 2012) yet only has 14 razor-sharp teeth in the lower jaw over which that force is distributed, potentially resulting in a bite stress that is significantly greater than any predator measured to date, relative to body size. Thus, we hypothesize that piranhas will have a wide range of bite forces but that these small predators possess some of the greatest size-specific bite forces and tooth stresses among animals.

In the present study our central aim is to calculate the maximum theoretical bite force and tooth stress of 11 piranha species from disparate feeding guilds, to advance our understanding of vertebrate bite mechanics. This is accomplished by first measuring the insertions of muscles and their tendons, relative to their position on the lower jaw. These data are used in a biomechanical model, with morphometrics and muscle architecture characteristics as inputs for the program PiranhaLever, which calculates maximum theoretical bite force. We then compute maximal bite forces over the total surface area of the dentition as well as the cutting edge of the tooth tip using 3D renderings of the teeth obtained from an orthodontic-grade, LiDAR scanner. Computational modeling provides an effective approach to reveal tooth bite stress in fishes and advances our understanding of the functional morphology and ecological implications of the jaws and teeth in piranhas and their kin. We predict that bite stresses will vary among species and that differences in performance correlate with ecology across piranhas that engage in everything from ectoparasitism (e.g., scale-feeding or lepidophagy) to varying degrees of omnivory and carnivory (feeding on fruits, seeds, insects, and fishes).

## Methods

### Piranha cranial anatomy

Freshly deceased specimens and thawed samples of recently deceased specimens for 11 piranha species (Table 1) were obtained from aquarium trade sources. In species for which there were multiple specimens, two individuals were used: a primary specimen and a surrogate specimen. The surrogate specimen was thoroughly dissected for reference of anatomical structures to subsequently mark the primary specimen’s skull anatomy and jaw features for quantification. In species for which there was only one specimen, the right side of the jaw was dissected and used as the surrogate for labeling the left side. This process was documented at each step with digital photographs with a 2 cm scale bar present for scale.

**Table 1:** Piranha species used in this study and their respective diet classifications

Species	Diet	Diet reference
*Catoprion mento*	Ectoparasite	Nico and Taphorn 1988
*Pygocentrus nattereri*	Carnivore	Nico and Taphorn 1988; doCarmo 2013
*Pygocentrus piraya*	Ectoparasite	Trindade and Juca-Chagas 2008
*Pygopristis denticulata*	Omnivore	Kolmann et al. 2024; Hemraj-Naraine, unpublished
*Serrasalmus altispinis*	Carnivore	Andrade et al. 2019, 2024
*Serrasalmus brandtii*	Carnivore	Pompeu 1999; Oliveira et al. 2004
*Serrasalmus eigenmanni*	Omnivore	Kolmann et al. 2024; Hemraj-Naraine, unpublished
*Serrasalmus elongatus*	Ectoparasite	Nico and Taphorn 1988
*Serrasalmus geryi*	Carnivore	Ropke 2008; doCarmo 2013
*Serrasalmus manueli*	Omnivore	Nico 1991; Dary et al. 2017
*Serrasalmus rhombeus*	Carnivore	Nico and Taphorn 1988

Full dissection of the surrogate specimen was completed prior to the primary specimen. Surrogate dissection began with the removal of the lips to expose the dentition. The circumorbital bones were removed revealing the adductor mandibulae (AM) complex. The adductor mandibulae is divided into the segmentum mandibularis (not quantified here because it does not contribute to bite force) and the segmentum facialis which is divided into three muscle subdivisions: the pars rictalis, the pars malaris*,* and the pars stegalis; the pars malaris being further divided into medial and lateral subdivisions (see Datovo and Vari 2013). The pars rictalis is a relatively small muscle that originates on the surface of the pars malaris lateral subdivision. The lateral portion of the pars rictalis inserts on the coronoid process of the dentary while the medial portion of the pars rictalis attaches to the medial surface of the dentary via a thin tendon. The pars malaris subdivisions constitute 80% or more of the adductor mandibulae complex in piranhas (Grubich et al. 2012; Huby et al. 2019). The lateral subdivision of the pars malaris originates from the preopercle while the medial subdivision originates from a ridge created by hypertrophication of the hyomandibula. The pars stegalis originates from underneath the hyomandibular ridge from which the pars malaris medial originates. The pars stegalis tendon fuses with the pars malaris tendon and, together, they insert on the medial surface of the dentary. This insertion point leads to increased mechanical advantage along the posterior half of the jaw, with the most posterior tooth having a mechanical advantage up to 1.5 (Grubich et al. 2012). A more thorough description of these muscles is available in Datovo and Castro (2012), Datovo and Vari (2013), and Huby et al. (2019).

Muscles were separated from their origins on the suspensorium to free the left half of the lower jaw for removal. The interoperculo-mandibular ligament (IOP-M) was severed to free the jaw from the opercular-series. An incision was made from the articular quadrate (AQ) joint along the medial surface of the dentary to the jaw symphysis to allow rostral-rotation of the jaw and musculature. This allowed the left half of the lower jaw to be removed from the surrogate fish with AM muscles intact.

Dissection of the primary specimen began with measuring and recording each specimen’s mass (g), standard length (SL; mm), and total length (TL; mm). Superficial dissection and removal of the lips and circumorbitals preceded isolation and identification of AM muscles. Utilizing surrogate specimen anatomy, tendon insertion points on the medial surface of the lower jaw were identified and marked with a black marker on the lateral jaw surface of the primary specimen for photographing and subsequent quantification. The left half of the lower jaw and its associated musculature were dissected away and removed as described for the surrogate dissection.

Employing a dissection probe along the epimysium, each muscle was isolated and identified. Individual muscles were separated at their respective insertions and detached from one another using a muscle probe. Muscle angles of pennation were measured with a protractor and recorded from three points: one halfway along the muscle body and dorsal to the tendon, one in-line with the tendon, and one halfway along the muscle body and ventral to the tendon. The mass of each AM muscle was measured using a Denver Instruments (TP323DS), Ohaus Scout Pro (SP 601), or Ohaus TR6RS (Ohaus Corp., Parsippany, NJ USA) digital balance (based on muscle size) and recorded.

### Morphometric data collection

**Fig. 2:**
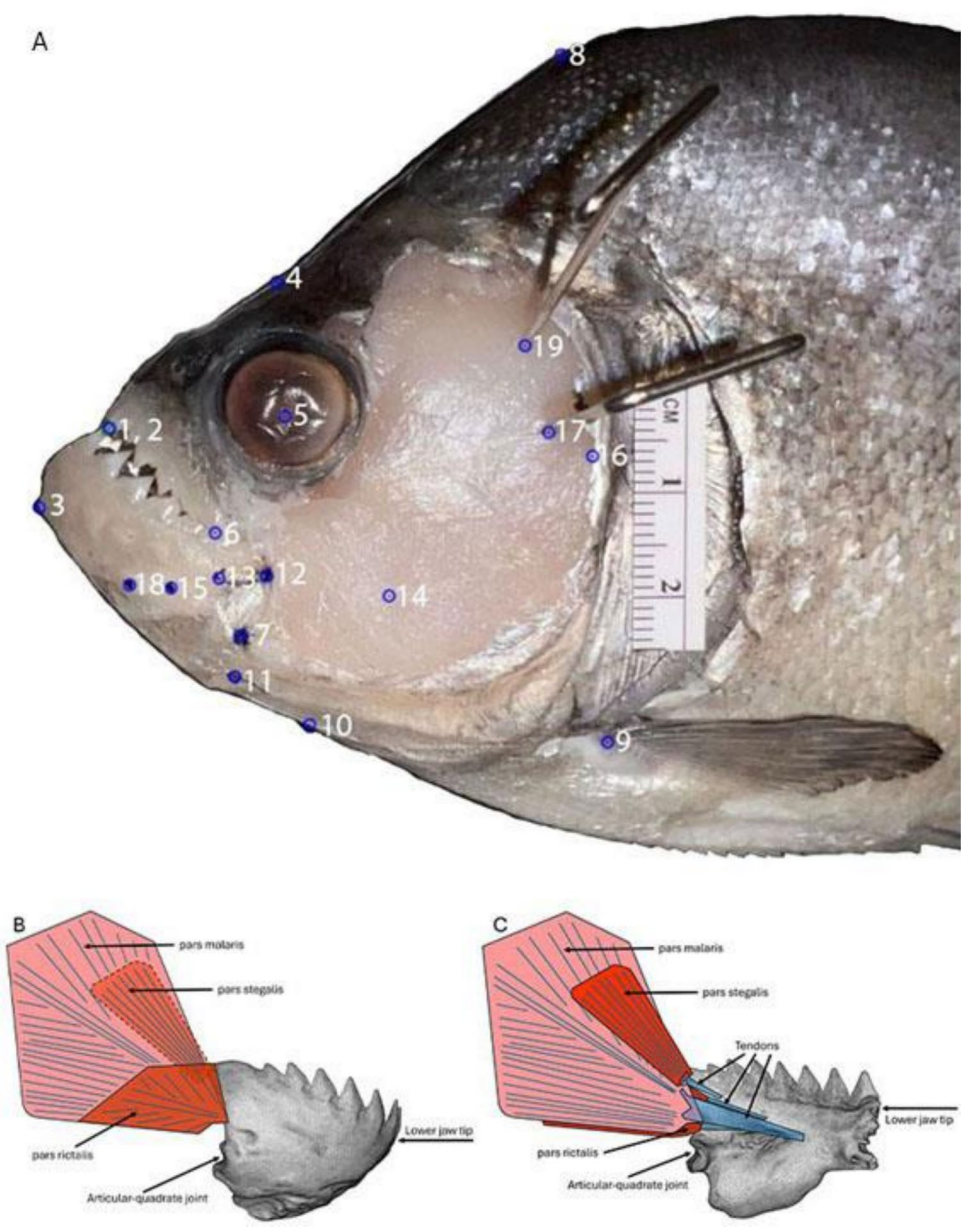
**A** Landmark points for importation into StereoMorph. (1) Tip of the rostral-most tooth of the upper jaw, (2) tip of the rostral-most tooth of the lower jaw, (3) rostral-most point on the ventral edge of the lower jaw, (4) a point on the dorsal edge of the skull above the eye, (5) center of the eye, (6) corner of the mouth, (7) articular-quadrate joint, (8) a point on the dorsal edge above the pectoral fin, (9) origin of the pectoral fin, (10) ventral edge of the skull, (11) insertion point of the interoperculo-mandibular ligament, (12) lateral insertion point of the pars rictalis tendon on the coronoid process, (13) medial insertion point of the pars rictalis tendon on the inside of the lower jaw, (14) origin of the pars rictalis muscle, (15) pars malaris (lateral and medial) tendon insertion point on the inside of the lower jaw, (16) pars malaris lateral origin on the suspensorium, (17) pars malaris medial origin on the suspensorium, (18) pars stegalis tendon insertion point on the inside of the lower jaw, (19) pars stegalis origin on the suspensorium. Specimen pictured is *Serrasalmus brandtii*. Lateral view **B** and medial view **C** of half of the lower jaw with associated muscles and tendons

Two-dimensional reconstruction was used as all muscle pathways are planar with morphometric markers used to model the fish jaw system (McCord and Westneat 2016; Olsen and Westneat 2016). The 2D landmarks of the piranha jaw system were obtained from a lateral image of the left side of the fish’s head (Fig. 2). Images were captured using a Canon EOS Rebel T6 or an iPhone 13 Pro, and landmarks were subsequently digitized using the R package StereoMorph (Olsen and Westneat 2015). Tendon lengths were acquired from images of the medial surface of the jaw after it had been dissected from the fish, also using StereoMorph.

### 3-D jaw rendering and tooth surface area quantification

**Fig. 3:**
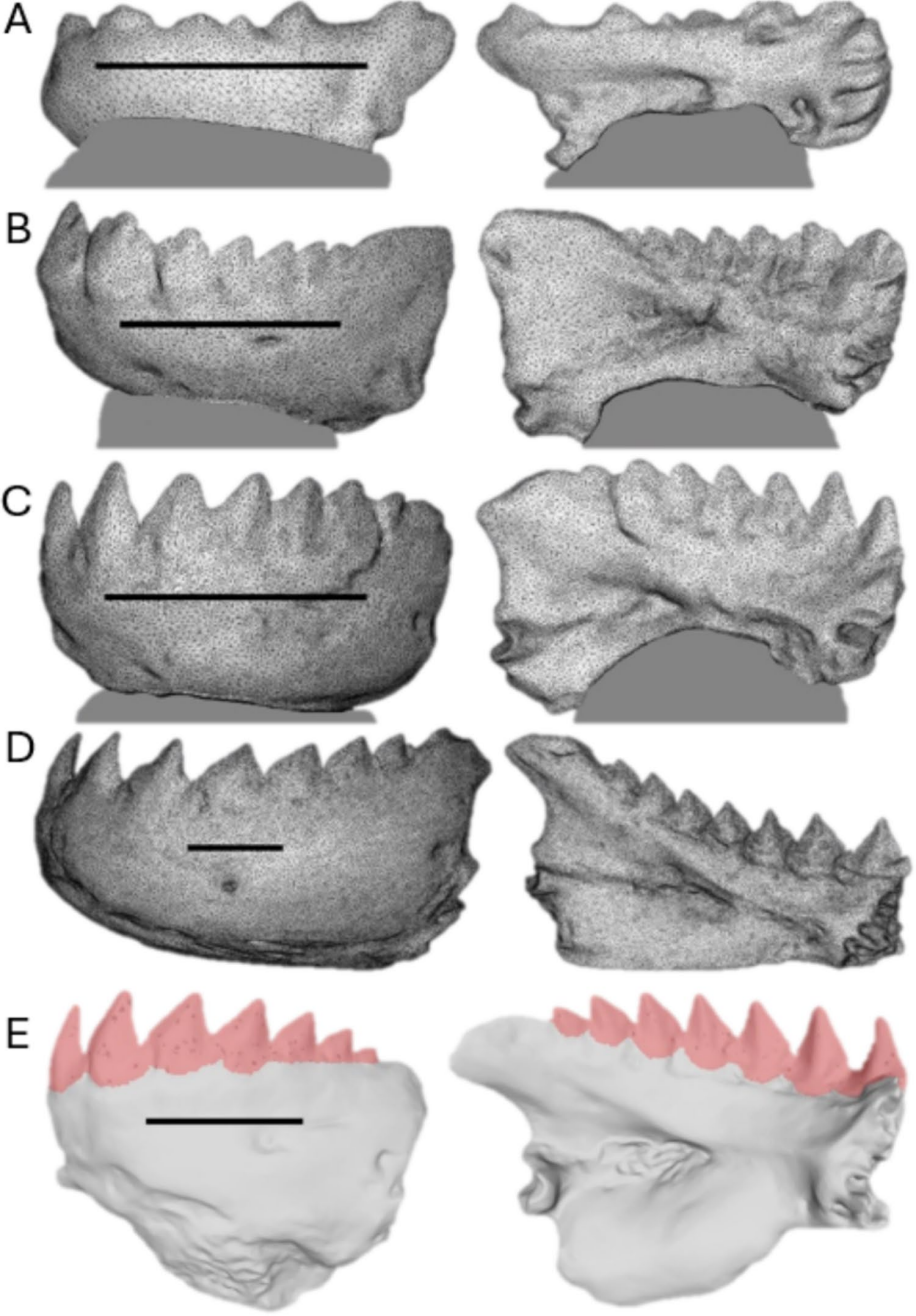
Lateral and medial views of 3D renderings of one half of the lower jaw used to quantify tooth surface areas in four of our specimens, **A** *Catoprion mento*, **B** *Pygopristis denticulata*, **C** *Pygocentrus piraya*, and **D** *Serrasalmus rhombeus*. A-C are mounted on grey clay. **E** Lateral and medial views of *Pygocentrus nattereri* 3D jaw rendering imported into MeshLab for quantification of total teeth (pictured in pink) and individual tooth surface areas. Scale bars are 1cm

The left half of the lower jaw was used for 3D renderings in nine of 11 species analyzed (Fig. 3, A-D). In two species, the left half was missing a tooth, so the right half was used instead. Each jaw-half was cleaned of any soft tissue and dried for 2 days. Due to reflectance, many required applications of a fine mist of grey spray paint prior to 3D scanning. Jaws too small to be handled during scanning (i.e., fingers would obstruct the scan) were embedded along their ventral surface into a block of modelling clay and set atop a dissection probe which allowed the jaw to be easily rotated. Larger jaws could be easily scanned by holding them. Three-dimensional rendering occurred with an Aoralscan3 orthodontic LiDAR scanner (Shining 3D Tech. Co., Ltd.; Hangzhou, China). The scanner created a 3D digital model of the jaws and teeth by constructing between 52,632 triangles for our smallest jaw (*C. mento*) and 200,176 triangles for our largest jaw (*S. rhombeus*), all of which were exported as.stl files for subsequent quantification of total teeth and individual tooth surface areas in MeshLab (Visual Computing Laboratory, Pisa, Italy).

Digitally rendered jaws were imported into MeshLab (version 2023.12) where all teeth were painstakingly enveloped using the surface area quantification tool (Fig. 3E). The images in Fig. 3E are simply to show the teeth in lateral and medial views. During surface area quantification in MeshLab, the entire 3D model could be rotated in three-planes to envelop the whole set of teeth, individual tooth, or 10% tooth tip as one contiguous surface area measurement. These files were also employed to measure the outlever-length of each tooth as the distance from the articular-quadrate joint to each tooth-tip to determine the mechanical advantage for each tooth along the jaw.

### Computational biomechanical modeling of piranha jaws

To calculate the forces of the adductor mandibulae muscles, the transfer of forces to the mandible via the jaw levers, and the total bite force and bite stress at each tooth, we developed the Mac desktop biomechanical modeling app PiranhaLever (Online Resource 1; app available at https://github.com/mwestneat). PiranhaLever is based on the app code for MandibLever (Westneat 2003, 2004), modified to include all adductor muscle subdivisions in piranhas and the calculations of force application and stress for all teeth along the lower jaw. The app first rotates each fish specimen to the horizontal axis and enables a set of batch processing (all specimens) and custom functions (individual specimen) to compute muscle length and force capacity at that length. For several specimens with the mouth slightly open, the lower jaw was rotated to close the mouth, through biomechanically computed jaw rotations, and the rotated coordinate sets were exported to use for final analyses.

Calculations by PiranhaLever include muscle lengths, muscle physiological cross-sectional area (PCSA) based on mass, length and pennation angles, lever dimensions (inlevers and outlever), muscle attachment angle, mechanical advantage, effective mechanical advantage, moment arm, maximum muscle force capacity assuming a peak force capacity of 300 kPa, contribution of each muscle subdivision to bite force at each tooth tip, the torque contribution of each muscle, and total summed bite force at each tooth tip for the muscles on one side of the head. The stress over each tooth surface was then calculated as total bite force divided by tooth surface area to yield bite stress in kPa (N/m2). We also computed the elevated stress at the tooth tip during initial tooth contact and puncture as bite force divided by the distal 10% of tooth area. PiranhaLever enables a pseudodynamic serial set of static calculations at multiple positions of the lower jaw, in units of 1 degree rotation, using a length tension curve computed from a Hill muscle model (Hill 1938), as implemented in MandibLever (Westneat 2003), where muscle force is lowest with jaws fully open at maximum muscle stretched length minF = (k-(k*maxV))/(k + maxV), and muscle force is maximal with the jaws closed maxF = (k-(k*minV))/(k + minV), where k = 0.25, maxV = 8 muscle lengths per second, and minV = 0.05 muscle lengths per second. These parameters can be varied in code, or by the user in the app interface. The full set of computations can be exported to a csv file for all fish specimens at any jaw position (Online Resource 1).

### Statistical analyses

Bite performance is relative to body size, so we also calculated a simple metric of mass-specific bite forces and tooth stresses by taking both metrics and dividing them by maximum body mass (kg) (Tables 2 and 3). These mass-specific metrics are useful for making physiological comparisons among both closely related species, like piranhas (Tables 2 and 3), as well as more inclusive comparisons across vertebrates (Table 4). While our calculated metrics do not adequately control for allometry or phylogenetic factors (and therefore should be interpreted with caution), they are more readily interpretable than independent contrasts or residual bite forces (see Erickson et al. 2012 for other methods). We used ordinary least squares regression (OLS) and the lm() function in R to examine trends between body size and mass-specific tooth stresses across vertebrates. We used ggplot2 to generate boxplots and biplots to visualize these data. All statistical analyses were performed in R version 4.4.2 (www.r-project.org).

**Table 2:** Bite forces from different piranha species, including mean bite force (averaged across all tooth positions) and mass-specific bite forces (maximum bite force divided by specimen mass)

Species	Mass (kg)	Standard length (mm)	Anterior bite force (N)	Posterior bite force (N)	Mean bite force (N)	Ant. mass-specific bite force (N/kg)	Post. mass-specific bite force (N/kg)
*Catoprion mento*	0.033	105.0	0.6	2.0	1.1	17.5	61.2
*Pygocentrus nattereri*	0.728	167.0	18.8	42.0	31.7	25.8	57.7
*Pygocentrus piraya*	0.056	223.0	16.9	38.8	29.6	300.0	687.3
*Pygopristis denticulata*	0.198	107.0	23.1	45.5	36.8	116.4	229.6
*Serrasalmus altispinis*	0.051	129.0	21.3	63.5	43.8	421.8	1257.4
*Serrasalmus brandtii*	0.233	190.0	32.7	100.9	69.0	140.5	433.4
*Serrasalmus eigenmanni*	0.102	140.0	19.7	50.8	36.6	193.3	498.5
*Serrasalmus elongatus*	0.042	134.0	11.1	50.4	25.4	266.2	1208.6
*Serrasalmus geryi*	0.040	115.0	12.0	29.1	21.2	299.3	725.7
*Serrasalmus manueli*	0.064	127.0	10.8	29.1	18.0	168.2	453.3
*Serrasalmus rhombeus*	1.222	298.0	53.5	117.7	89.7	43.8	96.3

**Table 3:** Bite stresses from different piranha species, including mean bite stress (averaged across all tooth positions) and total dental surface area

Species	Mass (kg)	Standard length (mm)	Anterior bite stress (kPa)	Posterior bite stress (kPa)	Mean bite stress (kPa)	Total dental surface area (mm^2^)
*Catoprion mento*	0.033	105.0	243.1	862.3	551.5	13.5
*Pygocentrus nattereri*	0.728	167.0	649.6	3308.9	2448.6	56.5
*Pygocentrus piraya*	0.056	223.0	1397.4	4881.8	4678.9	154.5
*Pygopristis denticulata*	0.198	107.0	1330.2	14667.3	10096.2	61.4
*Serrasalmus altispinis*	0.051	129.0	1486.2	11486.1	10360.5	63.4
*Serrasalmus brandtii*	0.233	190.0	1093.2	10139.5	6870.1	109.2
*Serrasalmus eigenmanni*	0.102	140.0	2039.4	10985.1	8075.9	43.4
*Serrasalmus elongatus*	0.042	134.0	943.4	16801.9	5119.0	47.7
*Serrasalmus geryi*	0.040	115.0	1074.3	7266.0	5422.8	41.9
*Serrasalmus manueli*	0.064	127.0	790.0	4165.2	2188.9	57.1
*Serrasalmus rhombeus*	1.222	298.0	744.2	3384.0	2435.6	325.8

**Table 4:** Comparison of feeding performance in vertebrates. Stress values reported here for piranhas include only the top 10% of the tooth tip surface area to better approximate methods used in the other studies listed. Some referenced studies did not report bite stresses alongside bite force values, so some of the bite stresses reported here reflect values gleaned from figures

Common name	Species	Body mass (kg)	Bite force (N)	Bite force/body mass (kg/N)	Bite stress (kPa)	Bite stress/body mass (kPa/kg)	Citation
Pike piranha	*Serrasalmus elongatus*	0.042	50.41	1208.77	167963	4027,890	This study
Red-spotted piranha	*Serrasalmus altispinis*	0.051	63.52	1257.79	114861	2274485	This study
Gery’s piranha	*Serrasalmus geryi*	0.040	29.06	724.69	72660	1811964	This study
Darwin’s finch	*Geospiza magnirostris*	0.033	44.00	1333.33	40000	1212121	Soons et al. 2015
Eigenmann’s piranha	*Serrasalmus eigenmanni*	0.102	50.75	498.04	109804	1077564	This study
Lobetoothed piranha	*Pygopristis denticulata*	0.198	45.47	229.64	146673	741148	This study
Manuel’s piranha	*Serrasalmus manueli*	0.064	29.07	452.85	41652	648781	This study
San Francisco piranha	*Pygocentrus piraya*	0.056	38.76	692.18	30545	545449	This study
Brandt’s piranha	*Serrasalmus brandtii*	0.233	100.89	433.37	101395	435545	This study
Wimple piranha	*Catoprion mento*	0.033	2.04	61.19	8623	258167	This study
Redbelly piranha	*Pygocentrus nattereri*	0.728	41.99	57.68	135451	186059	This study
Clevosaurus	*Clevosaurus cambrica*^†^	1.50	10.30	6.87	171230	114153	Chambi-Trowell et al. 2020
Jamaican fruit-eating bat	*Artibeus jamaicensis*	0.06	22.50	375.00	6450	107500	Dumont et al. 2005
Lesser short-nosed fruit bat	*Cynopterus brachyotis*	0.10	14.00	140.00	8710	87100	Dumont et al. 2005
Clevosaurus	*Clevosaurus hudsonix*^†^	1.50	14.90	9.93	106090	70727	Chambi-Trowell et al. 2020
Brown rat	*Rattus norvegicus*	0.50	N/A	N/A	25000	50000	Morris et al. 2022
Freshwater crocodile	*Crocodylus johnsoni*	43.00	1292.00	30.05	1871000	43512	Erickson et al. 2012
Grey squirrel	*Sciurus carolinensis*	0.750	N/A	N/A	25000	33333	Morris et al. 2022
Black piranha	*Serrasalmus rhombeus*	1.222	117.73	96.34	33840	27692	This study
Long-fingered lemur	*Daubentonia madagascariensis*	2.62	N/A	N/A	35000	13384	Morris et al. 2022
American alligator	*Alligator mississippiensis*	142.00	9452.00	66.56	1568000	11042	Erickson et al. 2012
Saltwater crocodile	*Crocodylus porosus*	272.00	16414.00	60.35	2473000	9092	Erickson et al. 2012
Black caiman	*Melanosuchus niger*	59.00	2696.00	45.69	509000	8627	Erickson et al. 2012
Orinoco crocodile	*Crocodylus intermedius*	182.00	6276.00	34.48	1388000	7626	Erickson et al. 2012
Beaver	*Castor canadensis*	50.00	N/A	N/A	40000	800	Morris et al. 2022
Spotted hyena	*Crocuta crocuta*	80.00	2014.00	25.18	46000	575	Tanner et al. 2008
Crested porcupine	*Hystrix cristata*	27.00	N/A	N/A	15000	556	Morris et al. 2022
T. rex	*Tyrannosaurus rex*^†^	5654.00	34522.00	6.11	2974000	526	Gignac and Erickson 2017
Placoderm	*Dunkleosteus terrelli*^†^	1000.00	5363.00	5.36	147000	147	Anderson and Westneat 2007

## Results

The forces of five jaw muscle subdivisions at their peak tension transmitted through piranha jaw levers with high mechanical advantage yielded the central result that piranhas produce extraordinarily strong, yet variable, bite forces. As predicted, bite forces and tooth stresses increased from anterior to posterior for all taxa. For non-size-corrected data, our largest species, the black piranha (*Serrasalmus rhombeus*), exhibited the greatest bite forces regardless of tooth position. The maximum bite force at the distal-most tooth reached 53.48 N, compared to a mean of 20.04 N across all piranha species. Force increased substantially at the proximal-most tooth, nearest the jaw joint, where *S. rhombeus’* maximum bite force was 138.47 N (compared to a mean of 51.92 N across all other species). The overall mean bite force across all species and all tooth positions was 36.62 N, peaking at 89.75 N in *S. rhombeus*. Note that *Serrasalmus elongatus* had the lowest mechanical advantage (closed position) at 0.14, while *Pygopristis denticulata* had the highest (0.47).

*Serrasalmus eigenmanni* (an omnivore) exhibited the greatest maximum bite stress at the distal-most tooth (as well as the next two anterior teeth), reaching 2,039.4 N/m^2^, compared to a mean of 1,071.9 N/m^2^ in other species. The greatest bite stress (in general) was observed in *Serrasalmus altispinis* (a carnivore), where the maximum at the proximal-most (i.e., nearest the jaw joint) tooth reached 44,378.7 N/m^2^, compared to a mean of 17,249.1 N/m^2^ for all piranhas. Averaged across all teeth, *S. altispinis* also had the greatest mean tooth stress, 10,360.5 N/m^2^, nearly twice the mean of all other piranhas (5,295.3 N/m^2^). However, at the fourth tooth position, the omnivorous *Pygopristis denticulata* exceeded both *S. eigenmanni* and *S. altispinis* with respect to maximum tooth stress (5,961.0 N/m^2^). This trend in greater tooth stress in more posterior teeth in *Pygopristis* continued until the sixth position, when *Serrasalmus elongatus*, a fin-feeder, exhibited the greatest tooth stress (16,801.9 N/m^2^).

*Catoprion*, an obligate ectoparasite, consistently exhibited the lowest values for bite force and tooth stress. Its mean bite force was 1.1 N, and its mean stress was 551.5 N/m^2^, an order of magnitude less than the mean stress observed across other piranhas. In terms of tooth surface area relative to body size (SA:SL ratio), *S. rhombeus* had the greatest values, with a maximum of 1.093, while *Catoprion* had the lowest across all measured parameters (0.129).

### Mass-specific bite forces & tooth stresses

Piranhas measured here ranged in body mass from 0.033 kg (*Catoprion mento*) to 1.222 kg (*Serrasalmus rhombeus*) (Table 2), with some of the smaller species producing the greatest mass-specific bite forces and tooth stresses. Mass-specific forces were greatest for posterior teeth, and posterior teeth were always smaller, with less surface area than anterior teeth, yielding high tooth stress values. *Serrasalmus altispinis* exhibited the greatest mass-specific bite forces overall (1,257.4 N/kg posterior; Table 2), followed by *S. elongatus* (1,208.6 N/kg posterior; Table 2). *P. piraya* and *S. geryi* also produced exceptionally high posterior mass-specific bite force values. As with the non-size-corrected performance data reported above, *Catoprion mento* had one of the lowest relative mass-specific bite forces at 61.2 N/kg posterior, barely exceeding *P. nattereri* (57.7 N/kg posterior; Table 2).

For tooth stresses, *Serrasalmus elongatus, S. altispinis,* and *Pygopristis denticulata* exhibited the greatest values, with posterior bite stresses exceeding 11,000–16,000 kPa and the latter two species also having mean bite stresses estimated above 10,000 kPa (Table 3). *Serrasalmus eigenmanni* and *S. brandtii* also produce consistently high posterior bite stress values, only slightly lower than that of *Pygopristis* and *S. altispinis*. *Catoprion mento* again exhibited the lowest tooth stress, while *Pygocentrus* and other *Serrasalmus* species occupy an intermediate range of performance values (Table 3).

### Comparison of tooth stresses among vertebrate top predators

**Fig. 4:**
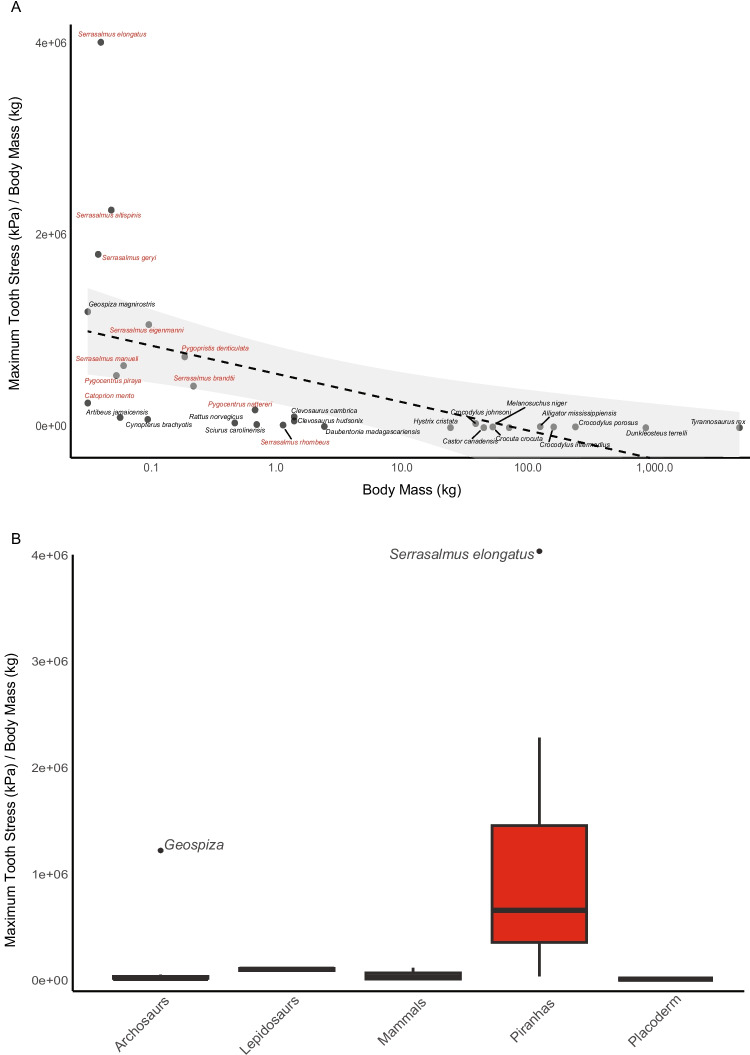
Mass-specific tooth stresses. **A** Regression of mass-specific tooth stresses across vertebrates against body mass. **B** Boxplot of mass-specific tooth stresses

Relative to other vertebrate predators for which bite stress values have been published, almost all piranhas tested here exceeded their counterparts when corrected for body mass (Table 4; Fig. 4). Apart from Darwin’s finch, all archosaurs, lepidosaurs, mammals, and the placoderm fell significantly below the mass-specific teeth stresses produced by nearly all our piranhas. Ten of the top eleven comparative bite stresses quantified were piranhas with Darwin’s finch occupying the other spot. The greatest mass-specific bite stress was exhibited by the pike piranha, *S. elongatus*, which produced 4,027,890 kPa/kg at the tip of its most proximate tooth, a value that is 7,658-times greater than the mass-specific bite stress of *T. rex* (526 kPa/kg; see Gignac and Erickson 2017) at its most procumbent maxillary tooth and almost 93-times greater than that of the highest crocodylian procumbent maxillary tooth value (freshwater crocodile 43,512 kPa/kg; see Erickson et al. 2012). Even the piranha with the weakest mass-specific bite stress (27,692 kPa/kg), *S. rhombeus*, yielded a tooth stress that is almost 53-times greater than that of *T. rex* and three-times greater than a saltwater crocodile (9,092 kPa/kg). Interestingly, the only two piranha species outside the top ten, *P. nattereri* and *S. rhombeus*, were also the two largest specimens we quantified, ranging from 22–37 times more massive than our smallest piranha, *C. mento*, to 3–5 times more massive than our third largest piranha, *S. brandtii*. This suggests there may be an allometric size-component to bite stress throughout ontogeny in piranhas, though this needs further investigation.

When comparing amongst other vertebrates for which teeth/beak stresses have been published, a relatively clear pattern emerges; smaller animals in each clade produce relatively greater bite stress (Fig. 4). Among archosaurs, only the Darwin’s finch exceeded the bite stress of some piranhas, and the finch is orders of magnitude smaller than the other archosaurs for which data exist (e.g., *T. rex* and crocodylians). In lepidosaurs, both species are extinct but are assumed to be relatively small (weighing approximately 1.5kg) and both produced reasonably high bite stresses, falling just outside our top ten. In mammals, the greatest values are in two small bat species, compared to the six larger mammals (e.g., beavers to hyenas) for which there are published bite stress data. Lastly, the placoderm, *D. terrelli*, was an enormous, formidable predator in its day, but relative to its body mass, appears to have produced reasonably weak jaw-edge stress during biting.

## Discussion

This study is the first to thoroughly describe the maximum bite stress of any vertebrate organism by integrating morphometrics, computational biomechanics, and orthodontic LiDAR technology. These data reveal that piranhas effectively translate significant jaw forces through their few, sharp teeth to create relative bite stresses that vastly exceed those of mega-predators, both extinct and extant. While *S. altispinis*, a known carnivore (Andrade et al. 2019, 2024), was the top-performer in overall mean bite stress across all teeth, several other piranha species exhibited remarkable bite performance relative to their known trophic ecologies. For example, the ectoparasitic pike piranha, *S. elongatus*, produced the greatest mass-specific bite stress ever calculated for a vertebrate organism, at 4,027,890 kPa/kg. We can draw multiple general conclusions from this study. First, piranha bite performance varied considerably among species, with some higher-level mesopredators like the black piranha having the greatest, absolute bite forces and stresses, while the lepidophagous wimple piranha had the weakest. Second, the three omnivorous species examined (*P. denticulata, S. eigenmanni,* and *S. manueli*) produced three of the top six greatest posterior tooth-tip bite stresses examined. And third, relative to body size, piranha tooth-tip bite stresses are orders of magnitude greater than most other vertebrates quantified to date. Computational models of bite force often underestimate real in vivo bite force. Our modeling here approaches in vivo measures of bite capacity in piranhas, yet remains conservative, suggesting that muscle mechanics and extreme bite performance warrant further study.

### Mesopredators, granivores, & ectoparasites, oh my! An ecological context for bite performance

Black piranhas (*Serrasalmus rhombeus*) consistently carve fins and flesh from prey fishes throughout their ontogeny (Winemiller 1989), with small, whole fishes comprising a greater proportion of their diet in mature individuals (Nico and Taphorn 1988). A combination of high bite force (Grubich et al. 2012) and high tooth stress (sharp teeth) may help explain the ubiquity of this species across South American aquatic habitats and likewise, their success as mesopredators. Similarly, red-belly piranhas (*Pygocentrus nattereri*) are also widespread in South America, albeit their ecology differs considerably from black piranhas, who hunt in small groups or individually, while red-bellies forage in large shoals (Winemiller 1989; Sazima and Machado 1990). However, when our feeding performance metrics were size-corrected (Fig. 4), piranha species such as the pike piranha (*Serrasalmus elongatus*) and lobetoothed piranha (*Pygopristis denticulata*) out-perform both black and red-belly piranhas.

*Serrasalmus elongatus* feeds primarily on fins, as well as scales, flesh, and smaller fishes (Nico and Taphorn 1988). The high tooth stresses observed in these fishes likely reflect the demands of rapidly and precisely excising the mineralized yet flexible fin rays of their prey, as piranhas like *Serrasalmus* generate intense, localized stresses around and between individual teeth to puncture and shear prey tissues (Rosen et al. 2025). In contrast, *Catoprion* exhibited the lowest bite force and tooth stresses of any fish in our dataset (perhaps due to their blunt teeth; Kolmann et al. 2018), but seemingly enough to dislodge scales from larger fishes (Huby et al. 2019). Although ectoparasites like pike piranhas and wimple piranhas (*Catoprion*) share similar behavioral ecologies, they lie on opposite ends of the performance spectrum. This disparity may be explained by differences in the material properties of their food and how their foods (scales, fins) are attached to prey (Janovetz 2005).

**Fig. 5:**
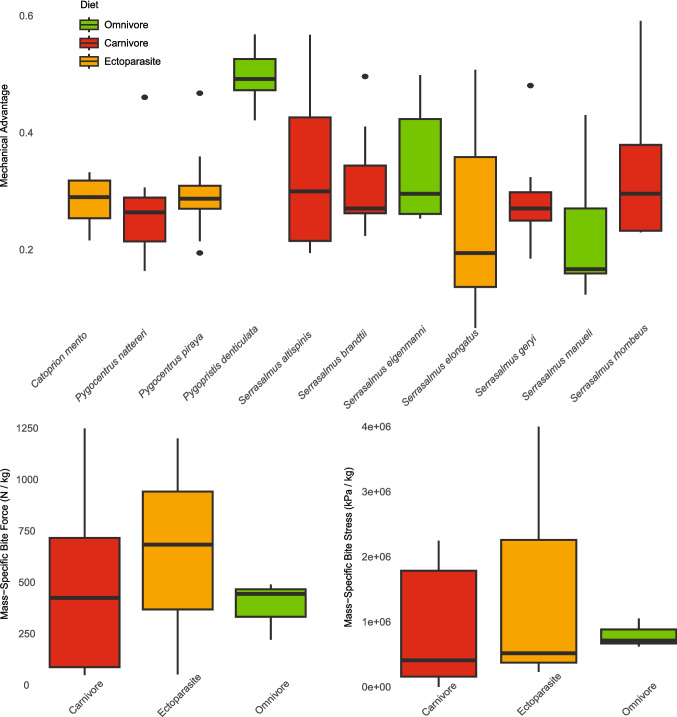
**A** Comparison of mechanical advantages (MA) among piranha species. Note the high MA in the omnivore and seed-eating species, *Pygopristis denticulata*. Mechanical advantages include the jaw-closing and muscle-specific jaw-closing mechanical advantage values. Mass-specific bite forces **B** and bite stresses **C** for three piranha feeding guilds

Contra to parasitic or carnivorous piranhas, *Pygopristis* is an omnivore that feeds on fruits, seeds, and occasional insects and scales (Kolmann et al. 2018, 2024; Hemraj-Naraine., unpublished). *Pygopristis* is also the only serrasalmid with pentacuspid teeth, with the multiple cusps presumably beneficial for trapping and then shearing tough prey materials (Lucas and Luke 1984; Abler 1992), such as seed husks. Despite having “blunter” teeth, *Pygopristis* produced high tooth stresses, not through excessive muscular force, but rather by having greater mechanical advantage (i.e., high leverage jaws; Fig. 5). Compared to black piranhas, which rely on large body size and correspondingly larger muscles to generate high bite forces, *Pygopristis* use optimal configurations of muscle in-levers to maximize transmission of force to prey.

High mass specific bite forces should be expected in granivorous piranhas since this trend is also seen in other seed-eating vertebrates such as finches (Grant and Grant 1989; Van der Meij and Bout 2006; Soons et al. 2015) and rodents (Hautier et al. 2011; Maestri et al. 2016). Seed-eating vertebrates typically require greater bite force because seeds often have tough outer shells or husks that must be cracked or crushed to access the nutritious content inside (Maestri et al. 2016). Adaptations for granivory in fishes are poorly understood; however, recent work (Rosen et al. 2025) demonstrates how granivores have blunter teeth which behave like a mortar and pestle, crushing outer seed coatings to access the interior. Likewise, frugivorous pacus (e.g., *Colossoma* and *Piaractus*) also have blunter teeth, suggesting that high surface area dentitions may be recurrent motifs in granivorous fishes (Rosen et al. 2025). Finally, *Serrasalmus eigenmanni*, another omnivorous, short-snouted piranha like *Pygopristis*, also had comparably high bite forces and greater jaw leverage – perhaps an emerging motif for omnivorous or herbivorous piranhas. Future work will be needed to determine if other seed-eating serrasalmids, characiforms, or other fishes in general, converge on similar feeding morphologies and mechanics.

### Piranhas as exceptional biters – comparisons with other studies

Piranhas generate tremendous bite stresses, relative to body size, on par with any vertebrate in Earth’s history. A closer look at the feeding behaviors of piranhas, relative to other vertebrate predators, reveals why: most piranha species surgically dismember prey, typically the ‘limbs’ (fins and fin bases; Nico and Taphorn 1988; Sazima and Pombal 1988; Winemiller 1990), rather than outright crush or twist carcasses apart. Bite wounds from piranhas, for example, are precise, clean cuts through flesh, sinew, and bone (Haddad and Sazima 2010).

Numerous researchers have examined how predators capture and incapacitate their prey and the most common metric for this interaction is bite force (for review, see Anderson et al. 2008). Bite force is useful in that it reveals what a predator is bringing to the predator–prey interaction and can shed light on proximate predator/prey performance (Huber et al. 2005; Herrel and Holanova 2008; Santana et al. 2010; Erickson et al. 2012) and/or ultimate evolutionary relationships (Arnold 1983; Christiansen and Wroe 2007; Irschick et al. 2008). Bite force also provides valuable predictive power for the maximum capabilities of predators, both extinct and extant (Erickson et al. 1996, 2003; Wroe et al. 2005). However, bite force fails at revealing what a predator is truly capable of when compared to the more telling *bite stress*. Very few researchers have attempted to quantify bite stress (sometimes referred to as *bite pressure* in the literature), and those who have were forced to use relatively basic methodologies to estimate tooth surface area because the technology for 3D scanning either did not exist (Snodgrass and Gilbert 1967), was in its infancy (Lucas 2004; Anderson and Westneat 2006; Tanner et al. 2008; Erickson et al. 2012), or was not employed (Gignac and Erickson 2015, 2017).

Comparing piranha bite stress to other taxa is challenging due to differences in methodologies across studies. Our conservative analysis accounts for the cumulative bite force across all lower jaw teeth, reflecting piranhas’ natural feeding behavior of engaging multiple teeth while headshaking to excise chunks of fins or flesh (Huskey and Kolmann, pers. obs.). In contrast, previous studies on crocodylians and *T. rex* analyzed only a small subset of teeth, often from the upper jaw (Erickson et al. 2012; Gignac and Erickson 2015, 2017), even though the lower jaw is the primary force generator during a bite. While upper jaw teeth may reactively contribute to bite success, this is only relevant for rigid prey like shells or bones, not the soft-bodied prey typically consumed by crocodylians and, likely, *T. rex*. Because soft-bodied prey conform to the shape of the jaws, nearly all lower jaw teeth are engaged (at least along one whole side of the jaw in crocodylians, for example), meaning bite stress should be calculated across the full tooth row. By analyzing only a few teeth, previous studies may have overestimated bite stress in these mega-predators. Our holistic approach provides a more ecologically relevant comparison, accounting for how force is naturally distributed across all teeth in a piranha jaw during biting.

Compared to the archosaurs for which bite stress has been calculated, piranhas out-perform nearly all groups when corrected for body size (Table 4). Of course, when considering absolute magnitude of bite force, enormous crocodylians and theropods produce bites that are unrivaled in nature (Rowe and Rayfield 2025). However, this is to be expected given their enormous body sizes. When scaled to correct for body size, the much smaller piranhas produce bite stresses that are nearly unrivaled. One exception is Darwin’s finch for which a bite stress value was gleaned from a figure (Soons et al. 2015; supplemental Fig. 003). This value was calculated from the upper jaw, so may not be ecologically relevant, but draws similarity to piranhas known to eat seeds given that Darwin’s finch is an obligate seed-eater. It likely uses the sharp edges of its beak to amplify bite force thereby producing bite stresses capable of cracking seed shells. Darwin’s finch is the only vertebrate, for which bite stresses have been quantified, to infiltrate the top ten of our analysis with piranha species occupying the other nine spots. Given the small size and sharp beaks of many seed-eating birds, it is likely that other bird taxa may produce size-corrected bite stresses that rival piranhas, but further studies that focus on lower jaw beak stresses are needed.

How do piranhas stack up against other charismatic predators, archosaurs aside? Methodologies vary widely, so our ability to compare among studies is limited. However, hyenas make for an excellent point of comparison, with skulls and jaws adapted for high-performance feeding, namely crushing bone (Binder and Van Valkenburgh 2000; Tanner et al. 2008). From 2D and pseudo-3D finite element modeling (FEM), researchers estimated that hyenas produce anywhere from 5.0–15.0 MPa in bite stresses (Rensenberger 1999; Tseng and DeSantis 2024). However, 15 MPa values from Rensenberger (1999) are recovered from tensile loads, so may not represent ecologically appropriate metrics for compressive biting. In absolute comparisons, piranhas produce lesser to equivalent bite stresses as hyenas, and by extension, exhibit similar performance to extant and extinct predators like cheetahs, lions, and *Hyaenodon* (all which fall within the 5 MPa maximum bite stresses; Tseng and DeSantis 2024) while weighing a fraction of these predators. However, these similarities fail when considering size-corrected bite stresses, in which piranhas far exceed their mammalian counterparts (Table 4).

## Conclusion

Ignoring the lore about piranhas, these diminutive fishes possess bite stresses that are nearly unrivaled by other top predators due to their hypertrophied skull and jaw bones, enormous jaw muscles amplified by increased biomechanical leverage, and relatively low numbers of razor-sharp teeth for delivery to their prey. Much exaggeration exists amongst the general public about piranhas as blood-thirsty attackers of people that reduce prey to skeletons in minutes. Most of these stories are unsupported by observation of piranhas in the wild. Even some researchers have made false claims about piranhas, such as attributing the evolution of the protective scales in *Arapaima gigas* to the bites of piranhas (for summary, see Huskey et al. 2020). Ignoring the hyperbole and conjecture, piranhas certainly represent one of nature’s most effective biters, possessing powerful jaws for their size and highly specialized dentition for efficiently dismantling prey.

## Supplementary Information

Below is the link to the electronic supplementary material.

Supplementary file1 (PDF 302 KB)

## Data Availability

PiranhaLever app available at https://github.com/mwestneat. Raw data available at 10.6084/m9.figshare.30842984.
