# Supplementary material for: Biomechanics, muscle modeling, and the elevated bite force and tooth stress of piranhas
Source: Naturwissenschaften. 2026 Feb 9;113(2):22. doi: 10.1007/s00114-026-02071-w (PMC12886308; doi:10.1007/s00114-026-02071-w)
Supplement: Supplementary file 1 — Supplementary file1 (PDF 302 KB) [file 114_2026_2071_MOESM1_ESM.pdf]

Supplemental Material for Biomechanics, muscle modeling, and the elevated bite force and tooth stress of piranhas

Journal Name: The Science of Nature

Authors: Steve Huskey[1], Keegan Fletcher, Matthew Kolmann, Serena Seiler, Gabrielle Kitchen, Devya Hemraj-Naraine, Ben Dinan, Mark W. Westneat

1. Department of Biological Sciences, Western Kentucky University, Bowling Green, KY 42101, United States

Corresponding Author: steve.huskey@wku.edu

Supplemental Methods:

Computational biomechanical modeling of piranha jaws

Calculations by PiranhaLever include muscle lengths, muscle physiological cross-sectional area (PCSA) based on mass, length and pennation angles, lever dimensions (inlevers and outlever), muscle attachment angle, mechanical advantage, effective mechanical advantage, moment arm, maximum muscle force capacity assuming a peak force capacity of 300 kPa, contribution of each muscle subdivision to bite force at each tooth tip, the torque contribution of each muscle, and total summed bite force at each tooth tip for the muscles on one side of the head. These parameters are all similar to those used in Westneat (2003) and more recent versions of the MandibLever software, and similar to those used by Huby et al. 2019 in their piranha jaw modeling. We used 300kPa as the peak force capacity of the jaw muscles (rather than 200 kPa as in Huby et al. 2019) because fish jaw muscles are usually near the maximum force capacity for vertebrate striated muscle, and because most lever models of fish jaws have been conservative, typically underestimating the *in vivo* bite forces measured from live animals exerting a maximal bite.

The stress over each tooth surface was then calculated as total bite force divided by tooth surface area to yield bite stress in kPa (kN/m^2). We also computed the elevated stress at the tooth tip during initial tooth contact and puncture as bite force divided by the distal 10% of tooth area. PiranhaLever enables a pseudodynamic serial set of static calculations at multiple positions of the lower jaw, in units of 1 degree rotation, using a length tension curve computed from a Hill muscle model (Hill 1938), as implemented in MandibLever (Westneat 2003), where muscle force is lowest with jaws fully open at maximum muscle stretched length minF = (k-(k*maxV))/(k+maxV), and muscle force is maximal with the jaws closed maxF = (k-(k*minV))/(k+minV), where k = 0.25, maxV = 8 muscle lengths per second, and minV = 0.05 muscle lengths per second. For this study we used the maximal force capacity of each muscle as the jaws are nearly fully closed, summed to give the total force at each tooth. We elected to use the summed forces of

just the single left side lateral muscles in computing bite force and tooth stress. It would be valid to double those forces to add the forces of the contralateral side muscles, particularly at the teeth tips. In fact, the current computed bite forces, when doubled, come a bit closer to the empirical measures of bite force by Grubich et al. 2012 and Huby et al. 2019. However, we were not certain that the tooth stresses would be properly calculated by doubling the force, as posterior teeth may not experience the full force of the contralateral side, so we chose the more conservative approach of modeling the left lateral system only. Important research remains to be done on the contractile physiology and peak force capacity of fish jaw muscles using strain gauges and FEA modeling to determine the tissue material properties upon which extreme bite forces depend.

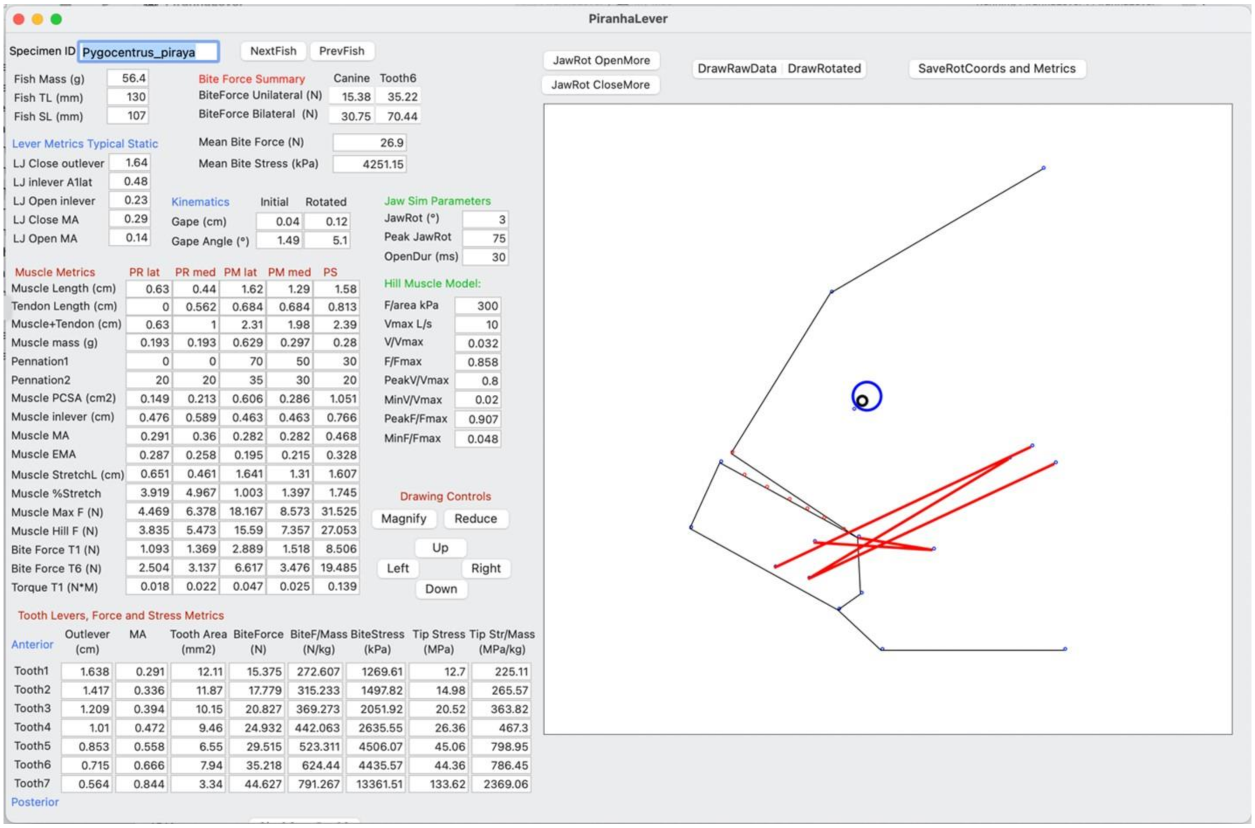

The PiranhaLever app user interface, illustrating the user tools for calculating biomechanical lever and muscle function, and for computing piranha bite force and tooth stresses. The PiranhaLever Mac desktop or laptop app is available at https://github.com/mwestneat/PiranhaLever.

Supplemental Results:

The raw data collected for analysis in PiranhaLever are available in a repository on FigShare at https://doi.org/10.6084/m9.figshare.30842984. These files include the piranha jaw lever coordinates, the muscle mass, tendon, and pennation data, and the data on tooth surface areas.
